# Multilevel interactions between native and ectopic isoprenoid pathways affect global metabolism in rice

**DOI:** 10.1007/s11248-022-00299-6

**Published:** 2022-02-24

**Authors:** Lucía Pérez, Rui Alves, Laura Perez-Fons, Alfonso Albacete, Gemma Farré, Erika Soto, Ester Vilaprinyó, Cristina Martínez-Andújar, Oriol Basallo, Paul D. Fraser, Vicente Medina, Changfu Zhu, Teresa Capell, Paul Christou

**Affiliations:** 1 Department of Plant Production and Forestry Science, School of Agrifood and Forestry Science and Engineering (ETSEA), University of Lleida-Agrotecnio Center, Av. Alcalde Rovira Roure 191, 25198 Lleida, Spain; 2 Departament de Cienciès Mèdiques Bàsiques, Universitat de Lleida, Lleida, Spain; 3 School of Biological Sciences, Royal Holloway University of London, Egham Hill, UK; 4 Departament of Plant Nutrition, Center of Edaphology and Applied Biology of the Segura (CEBAS), Consejo Superior de Investigaciones Científicas (CSIC), Campus Universitario de Espinardo, 30100 Murcia, Espinardo Spain; 5 Department of Plant Production and Agrotechnology, Institute for Agri-Food Research and Development of Murcia, Murcia, La Alberca Spain; 6 Department of Chemistry, University of Lleida-Agrotecnio Center, Lleida, Spain; 7 IRBLleida, Lleida, Catalunya Spain; 8 Catalan Institute for Research and Advanced Studies (ICREA), Barcelona, Spain

**Keywords:** Engineered plants, Isoprenoids, Metabolomics, Mevalonate pathway, *Oryza sativa*

## Abstract

Isoprenoids are natural products derived from isopentenyl diphosphate (IPP) and dimethylallyl diphosphate (DMAPP). In plants, these precursors are synthesized via the cytosolic mevalonate (MVA) and plastidial methylerythritol phosphate (MEP) pathways. The regulation of these pathways must therefore be understood in detail to develop effective strategies for isoprenoid metabolic engineering. We hypothesized that the strict regulation of the native MVA pathway could be circumvented by expressing an ectopic plastidial MVA pathway that increases the accumulation of IPP and DMAPP in plastids. We therefore introduced genes encoding the plastid-targeted enzymes HMGS, tHMGR, MK, PMK and MVD and the nuclear-targeted transcription factor WR1 into rice and evaluated the impact of their endosperm-specific expression on (1) endogenous metabolism at the transcriptomic and metabolomic levels, (2) the synthesis of phytohormones, carbohydrates and fatty acids, and (3) the macroscopic phenotype including seed morphology. We found that the ectopic plastidial MVA pathway enhanced the expression of endogenous cytosolic MVA pathway genes while suppressing the native plastidial MEP pathway, increasing the production of certain sterols and tocopherols. Plants carrying the ectopic MVA pathway only survived if WR1 was also expressed to replenish the plastid acetyl-CoA pool. The transgenic plants produced higher levels of fatty acids, abscisic acid, gibberellins and lutein, reflecting crosstalk between phytohormones and secondary metabolism.

**Supplementary Information:**

The online version contains supplementary material available at . 10.1007/s11248-022-00299-6

## Introduction

**Fig. 1:**
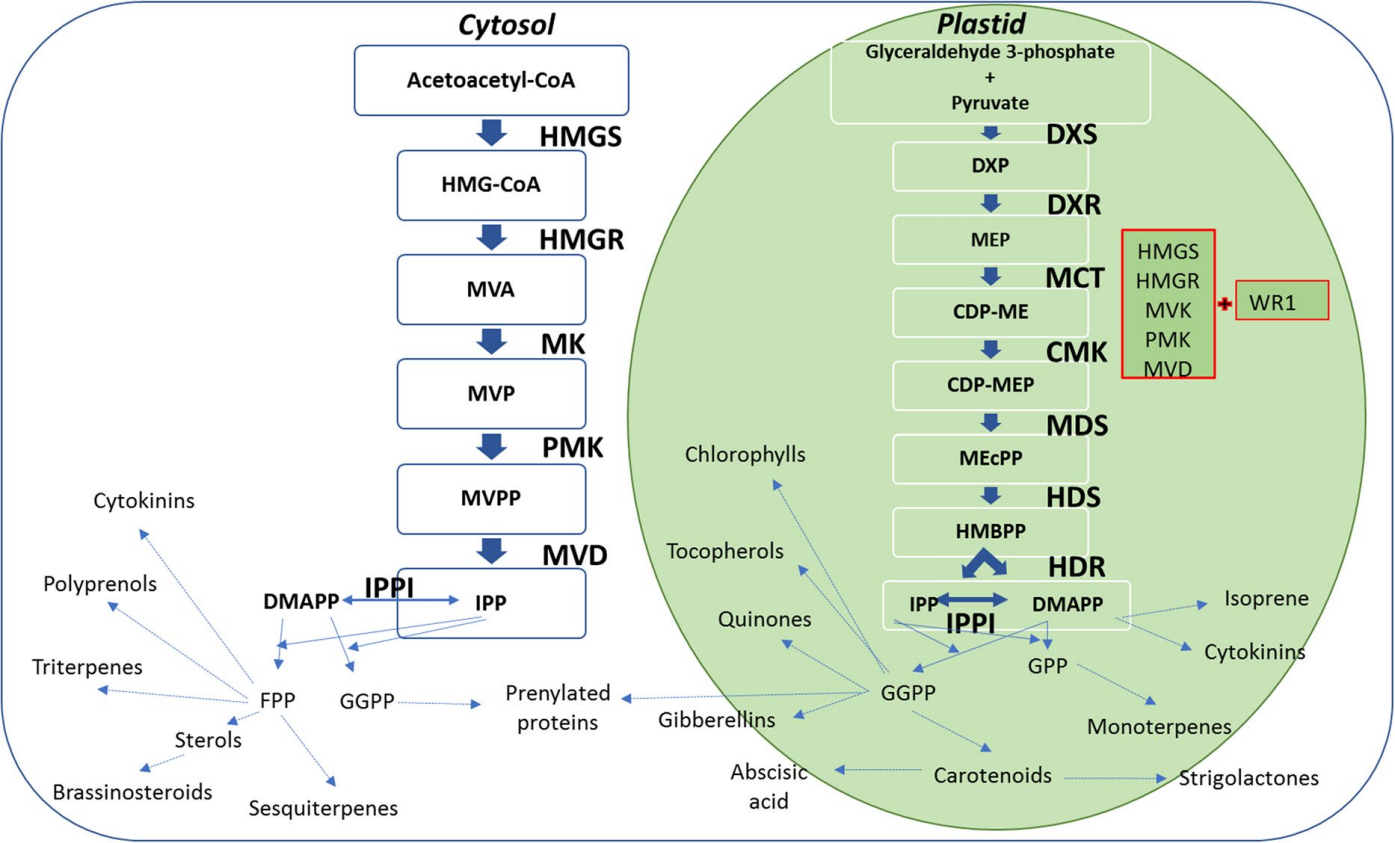
Steps in the isoprenoid biosynthesis pathway and representation of the different genetic approaches we used to express a complete or partial ectopic MVA pathway (red boxes). Abbreviations: HMGS, 3-hydroxy-3-methylglutaryl-CoA synthase; HMGR, 3-hydroxy-3-methylglutaryl-CoA reductase; MK, mevalonate kinase; PMK, 5-phosphomevalonate decarboxylase; MVD, mevalonate diphosphate decarboxylase; IPPI, isopentenyl diphosphate isomerase; FPP, farnesyl diphosphate; GGPP, geranylgeranyl diphosphate; DMAPP, dimethylallyl diphosphate; IPP, isopentenyl pyrophosphate; HMG-CoA, 3-hydroxy-3-methylglutaryl-CoA; MVA, mevalonic acid; MVP, mevalonate-3-phosphate; MVPP, mevalonate- 3,5-diphosphate; DXS, 1-deoxy-d-xylulose-5-phosphate synthase; DXR, 1-deoxy-d-xylulose-5-phosphate reductoisomerase; MCT, 4-diphosphocytidyl-2C-methyl-d-erythritol 4-phosphate synthase; CMK, 4-(cytidine-5′-diphospho)-2-C-methyl-d-erythritol kinase; MDS, 2C-methyl-d-erythritol 2,4-cyclodiphosphate synthase; HDS, 4-hydroxy-3-methylbut-2-enyl diphosphate synthase; HDR, 1-hydroxy-2-methyl-2-(E)-butenyl 4-diphosphate reductase; MEP, 2C-methylerythritol 4-phosphate; CDP-ME, 4-(cytidine-5′-diphospho)-2-C-methyl-d-erythritol; CDP-MEP, 4-diphosphocytidyl-2C-methyl-d-erythritol 4-phosphate; ME-cPP, 2C-methyl-d-erythritol 2,4-cyclodiphosphate; HMBPP, (E)-4-hydroxy-3-methyl-but-2-enyl pyrophosphate; WR1, WRINKLED 1

Isoprenoids are the largest family of natural products, with at least 50,000 different structures identified thus far (Vranova et al. 2013; Farré et al. 2015). These metabolites are essential for many fundamental biological processes, and organisms that cannot synthesize them are therefore obligate parasites (Boucher and Doolittle 2000). All isoprenoids are assembled from the precursors isopentenyl diphosphate (IPP) and dimethylallyl diphosphate (DMAPP), which are synthesized via the mevalonate (MVA) or methylerythritol phosphate (MEP) pathways (Fig. 1). Unlike most organisms, plants possess both pathways and thus provide insight into cross-pathway interactions during the regulation of isoprenoid metabolism (Kovacs et al. 2002).

In plants, the MVA pathway produces isoprenoids in the cytosol and mitochondria whereas the MEP pathway produces isoprenoids in the plastids. The MVA pathway comprises six enzymatic steps that convert acetyl-CoA into IPP, as well as the enzyme IPP isomerase (IPPI) which interconverts IPP and DMAPP and maintains equilibrium between these two molecules. Acetoacetyl-CoA thiolase (ACAT) is also required, but is not considered as an exclusive MVA pathway enzyme because its function is common to other processes such as fatty acid β-oxidation (Pereto et al. 2005), and it is present in the cytosol and peroxisomes in addition to plastids (Carrie et al. 2007; Wentzinger et al. 2012). The MEP pathway comprises eight enzymatic steps that convert glyceraldehyde-3-phosphate and pyruvic acid into IPP and DMAPP in a 5:1 ratio (Cunningham et al. 2000). Substantial cross-talk between the two pathways has been reported (Bouvier et al. 2005; Vrarona et al. 2012). Inhibition of the MVA and MEP pathways using specific inhibitors (mevinolin and fosmidomycin, respectively) revealed the exchange of metabolites between the pathways to provide substrates for sterol synthesis downstream of the inhibited pathway (Hemmerlin et al. 2003) but this is probably insufficient to compensate for the loss of flux caused by non-functional enzymes in either pathway (Ishiguro et al. 2010; Vranova et al. 2013).

The MVA pathway is subject to complex multilevel feedback regulation (transcriptional, post-transcriptional, translational and post-translational) and an extra level of regulation is conferred by multiple enzyme isoforms that are differentially expressed according to the environmental conditions (Hemmerlin et al. 2012; 2013). One of the key regulatory factors is light, which inhibits the transcription of MVA pathway genes such that isoprenoid synthesis occurs mostly in the dark (Vranova et al. 2013). The MVA pathway is also responsive to osmotic stress, dehydration, temperature changes, UV light, bacterial pathogens, fungal elicitors, herbivory, wounding and mycorrhiza (Cordoba et al. 2009). A high AMP/ATP ratio increases the activity of an AMP-activated protein kinase that phosphorylates MVA pathway enzymes to block their activity (Burg and Espenshade 2011). IPP and DMAPP also regulate the expression of MVA and MEP pathway genes through feedback inhibition (Banerjee et al. 2013).

The rate-limiting enzyme in the MVA pathway is 3-hydroxy-3-methyl-glutaryl coenzyme A reductase (HMGR), which is regulated at multiple levels (Ferrero et al. 2015; Tsuruta et al. 2009; Ma et al. 2011). It is inhibited by both of its substrates: HMG-CoA and acetoacetyl-CoA (Wang et al. 2012). Proteins anchored in the endoplasmic reticulum membrane interact with the HMGR membrane-spanning domain (Hemmerlin 2013) to regulate the catalytic domain in the cytosol (Burg and Espenshade 2011). Attempts to increase flux through the MVA pathway by increasing precursor availability have been unsuccessful due to the complex regulation of HMGR (Alper et al. 2005; Zhao et al. 2013). However, the inhibition of this enzyme can be lifted partly by expressing a truncated version (tHMGR) lacking regulatory motifs in the membrane-spanning domain (Harker et al. 2003).

Disruption of either the MVA or MEP pathway in plants causes deleterious phenotypes such as sterility and abnormal development because isoprenoid precursors are required to synthesize many important phytohormones, including auxins (Woodward et al. 2005), ethylene (Wang et al. 2002; Erb et al. 2012), jasmonic and salicylic acid (Browse. 2009; An and Mou 2011), cytokinins (Frebort et al. 2011), abscisic acid (ABA) (Nambara and Marion-Poll 2005) and gibberellins (Hedden and Thomas 2012). It is therefore necessary to understand the regulation of these pathways in order to engineer isoprenoid synthesis without pleiotropic effects. Components of the yeast (*Saccharomyces cerevisiae*) MVA pathway have been introduced into tobacco (*Nicotiana tabacum*) plastids by plastid transformation to increase flux in the MVA pathway, causing a significant change in the squalene content of tobacco leaves but no discernible macroscopic phenotype (Kumar et al. 2012). However, the study did not measure precursor levels, phytohormones or primary metabolites such as sugars and fatty acids, so the wider impact of the ectopic pathway is unknown.

A significant challenge that influences metabolic engineering in vegetative organs such as leaves is that any ectopic pathway must coexist with essential metabolic activity during photosynthesis and carbon assimilation. In contrast, mature cereal endosperm is almost completely metabolically inactive and provides a blank canvas for metabolic engineering, as we previously demonstrated by combinatorial transformation to develop a maize (*Zea mays*) population with diverse carotenoid and ketocarotenoid profiles in the endosperm (Zhu et al. 2008, 2009). Engineering the accumulation of IPP/DMAPP in cereal seeds could therefore provide a universal isoprenoid chassis, allowing the conversion of these precursors into specific target compounds by the endosperm-specific expression of downstream enzymes. Synergy between ectopic and endogenous MVA and MEP pathway components in bacteria produces higher levels of IPP and DMAPP than a single enhanced pathway (Yang et al. 2016). We therefore hypothesized that the strict regulation of the endogenous cytosolic MVA pathway in plants could be overcome by expressing an ectopic plastidial MVA pathway, potentially increasing the production of IPP and DMAPP, although this might trigger knock on effects on other pathways because the MVA pathway draws on the limited plastidial pool of acetyl-CoA (Cernac and Benning 2004). We further hypothesized that network analysis might help to explain the multilevel interactions that translate genetic modifications into metabolic phenotypes. The plastid acetyl-CoA pool can be enhanced by expressing rice (*Oryza sativa*) homologs of the *Arabidopsis thaliana* transcription factor WRINKLED1 (WR1) to induce the expression of genes related to plastid glycolysis and fatty acid biosynthesis (Cernac and Benning 2004). The overexpression of *WR1* in *A. thaliana* seeds upregulated the fatty acid biosynthesis pathway by directly activating genes required for the synthesis and storage of triacylglycerols (Maeo et al. 2009). Similarly, the overexpression of *ZmWri1a* in maize seeds increased the content of fatty acids, amino acids and certain organic acids (Pouvreau et al. 2011). The expression of WR1 proteins has also been shown to boost fatty acid biosynthesis in *Nicotiana benthamiana* (Grimberg et al. 2015), *Brachypodium distachyon* (Yang et al. 2015) and potato (*Solanum tuberosum*) (Hofvander et al. 2016), with corresponding changes in glycolysis and carbohydrate metabolism.

To understand the effects of an ectopic MVA pathway in plastids, we constructed an ectopic MVA pathway and introduced the corresponding transgenes into rice, controlled by endosperm-specific promoters (Fig. 1). We expressed a complete ectopic MVA pathway (HMGS, tHMGR, MK, PMK and MVD) and a complete ectopic MVA pathway plus WR1 in separate experiments. We determined the effect of the ectopic pathways by quantifying the transcripts of MVA and MEP pathway genes and the levels of various isoprenoids as proxies for IPP/DMAPP, which are difficult to measure directly because of their rapid turnover. We anticipated changes in the expression of endogenous MVA and MEP pathway genes owing to cross-regulation with the ectopic pathway. We also hypothesized that the ectopic pathway would increase the levels of total isoprenoids and squalene. To investigate potential pleotropic effects of the ectopic pathway, we measured the levels of phytohormones and primary metabolites. Our results provide insight into the regulation of isoprenoid metabolism in rice and will allow the development of rational predictive strategies for isoprenoid engineering in plants.

## Materials and methods

### Construction of transformation vectors

The six transgenes (*BjHMGS, tHMGR, CrMK, CrPMK, CrMVD* and *OsWR1*) were housed in three separate expression cassettes driven by endosperm-specific promoters. The five enzymes in the MVA pathway were provided with a transit peptide to direct them to the plastid whereas WR1 was directed to the nucleus by its intrinsic nuclear localization signal (NLS). The *Brassica juncea* HMGS gene (*BjHMGS*) was placed under the control of the barley (*Hordeum vulgare*) D-hordein promoter and *Agrobacterium tumefaciens* nopaline synthase terminator (*nos*). The *A. thaliana tHMGR* gene was placed under the control of the wheat (*Triticum aestivum*) low-molecular-weight glutenin promoter and the rice ADPGPP terminator. The *Catharanthus roseus MK* gene (*CrMK*) was placed under the control of the barley D-hordein promoter and *nos* terminator. The *C. roseus PMK* gene (*CrPMK*) was placed under the control of the maize γ-zein promoter (GZ63) and *nos* terminator. The *C. roseus MVD* gene (*CrMVD*) was placed under the control of the rice prolamin promoter (RP5) and *nos* terminator. Finally, the rice *WR1* gene (*OsWR1*) was placed under the control of the RP5 promoter and *nos* terminator. All sequences were codon optimized for rice and pre-assembled by GenScript (Piscataway, NJ, USA) on three vectors. The first vector contained the first three genes of the MVA pathway (*BjHMGS:tHMGR:CrMK*), the second vector contained the two last genes of the MVA pathway (*CrPMK:CrMVD*) and the third vector contained *OsWR1*. An additional plasmid containing the hygromycin phosphotransferase (*hpt*) selectable marker gene (Christou et al. 1991) under the control of the constitutive cauliflower mosaic virus 35S promoter and *nos* terminator was used to select the transgenic rice plants.

### Rice transformation and recovery of plants

Seven-day-old mature zygotic rice embryos (*Oryza sativa* cv. EYI105) were transferred to osmotic medium (MS medium supplemented with 0.3 g/L casein hydrolysate, 0.5 g/L proline, 72.8 g/L mannitol and 30 g/L sucrose) 4 h before bombardment with 10 mg gold particles coated with the transformation vectors. The expression vectors were provided as a three-fold molar excess over the selectable marker as previously described (Sudhakar et al. 1998; Valdez et al. 1998) resulting in a molar ratio of 3:3:3:1 for the BjHMGS*:tHMGR:CrMK* + *CrPMK:CrMVD* + *OsWR1* + *hpt* transformation. The embryos were returned to osmotic medium for 12 h before selection on MS medium supplemented with 0.3 g/L casein, 0.5 g/L proline, 30 g/L sucrose, 50 mg/L hygromycin and 2.5 mg/L 2,4-dichlorophenoxyacetic acid in the dark for 2–3 weeks. Callus was maintained on selective medium for 6 weeks with sub-culturing every 2 weeks as previously described (Farré et al. 2012). Transgenic plantlets were regenerated and hardened off in soil. Genomic DNA was isolated from the callus and leaves of regenerated plants by phenol extraction and ethanol precipitation (Bassie et al. 2008; Kang and Yang 2004). The presence of the *BjHMGS, tHMGR, CrMK, CrPMK, CrMVD* and *OsWR1* transgenes was confirmed by PCR using the primers listed in Supplementary Table 1.

### Phytohormone analysis

We analyzed the cytokinins *trans*-zeatin, zeatin riboside and isopentenyl adenine; the gibberellins GA1, GA3 and GA4; the auxin indole-3-acetic acid (IAA); ABA; salicylic acid; jasmonic acid; and the ethylene precursor 1-aminocyclopropane-1-carboxylic acid (ACC) as previously described (Albacete et al. 2008) with some modifications. Briefly, 0.1 g of fresh leaf material was homogenized in liquid nitrogen and immersed in 1 mL 80:20 (v/v) methanol/water at –20 °C. Solids were separated by centrifugation (20,000 × g, 15 min, 4 °C) and re-extracted for 30 min at 4 °C as above. Pooled supernatants were separated using Sep-Pak C18 Plus cartridges (Waters, Milford, MA, USA) to remove interfering lipids and pigments, and evaporated at 40 °C under vacuum to near dryness. The residue was dissolved in 0.5 mL 20:80 (v/v) methanol/water using an ultrasonic bath. The dissolved samples were passed through 0.22-µm Millex nylon filters (Millipore, Bedford, MA, USA). The filtered extract (10 µL) was injected into an Accela Series ultra-high-performance liquid chromatography (UHPLC) system coupled to an Exactive mass spectrometer (Thermo Fisher Scientific, Waltham, MA, USA) via a heated electrospray ionization (HESI) interface. Mass spectra were obtained using Xcalibur v2.2 (Thermo Fisher Scientific). For quantification, calibration curves were constructed for each phytohormone (1, 10, 50 and 100 µg L^−1^) and corrected for 10 µg L^−1^ deuterated internal standards. We achieved 92–95% target compound recovery.

### Phenotypic analysis

The transgenic rice lines were analyzed after 12 weeks growing in soil. We counted the number of leaves and measured the height of the plants from the base of the stem to the maximum extension of the flag leaf. The leaf chlorophyll content was measured with a SPAD meter at six points on the last expanded leaf of 10 plants (biological replicates). The length and maximum width of the last expanded leaf were multiplied and a correction factor of 0.75 was used to calculate the area, accounting for the leaf shape (Tsunoda 1962; Murata et al., 1967). Images of five plants (biological replicates) were also acquired at 12 weeks to compare the height and state of leaf senescence in the transgenic lines to wild-type controls.

### Gene expression analysis

Total seed RNA was isolated using the RNeasy Plant Mini Kit (Qiagen, Hilden, Germany) and DNA was digested with DNase I from the RNase-free DNase Set (Qiagen). Total RNA was quantified using a Nanodrop 1000 spectrophotometer (Thermo Fisher Scientific) and 2 μg of total RNA was used as a template for first strand cDNA synthesis with Quantitech reverse transcriptase (Qiagen) in a 20-μL reaction volume. Real-time qRT-PCR was carried out on a CFX96 system (Bio-Rad, Hercules, CA, USA) using 20-μL mixtures containing 5 ng cDNA, 1 × iQ SYBR Green Supermix and 0.5 μM of the forward and reverse primers designed for the transgenes *BjHMGS, tHMGR, CrMK, CrPMK, CrMVD* and *OsWR1*, and the endogenous MVA and MEP pathway genes *OsHMGS, OsHMGR, OsMK, OsPMK, OsMVD, OsDXS, OsDXR, OsMCT, OsCMK, OsMDS, OsHDS*, *OsHDR* and *OsIPPI* (Supplementary Table 1). Serial dilutions of cDNA (80–0.0256 ng) were used to generate standard curves for each gene. PCR was carried out in triplicate using 96-well optical reaction plates. Each value is presented as the mean of three biological replicates ± standard deviation (SD) (Supplementary Table 2). Amplification efficiencies were compared by plotting the ΔCt values of different primer combinations of serial dilutions against the log of starting template concentrations using CFX96 software. The rice housekeeping *OsActin1* (ABF98567.1) was used as an internal control.

A GenBank search to select endogenous rice MVA pathway genes for analysis revealed three isoforms of *OsHMGS* (*OsHMGS*1–3), three isoforms of *OsHMGR* (*OsHMGR*1–3), one isoform of *OsMK*, two alternative splice variants of *OsPMK* (*OsPMKa* and *OsPMKb*) and two isoforms of *OsMVD* (*OsMVD1* and *OsMVD2*). Similarly, for the MEP pathway, we identified three isoforms of *OsDXS* (*OsDXS*1–3), one isoform each of *OsDXR*, *OsMCT*, *OsCMK*, *OsMDS* and *OsHDS*, two isoforms of *OsHDR* (*OsHDR1* and *OsHDR2*) and two isoforms of *OsIPPI* (*OsIPPI1* and *OsIPPI2*). The accession numbers for all variants in both pathways are provided in Supplementary Table 3.

### Carotenoid extraction and analysis

Dried seeds were ground to a fine powder using a mortar and pestle, and 30 mg of powder was used for carotenoid extraction and analysis (Bligh and Dyer 1959). Briefly, we mixed the powder with 400 μL of methanol and 800 μL of chloroform for 1 h in the dark before adding 400 μL of water. The mixture was centrifuged at 13,000 × g for 5 min and the organic layer containing carotenoids was collected, dried under a stream of N_2_ and stored at –20 °C. The dried extracts were dissolved in 50 μL ethyl acetate and centrifuged at 13,000 × g for 10 min. We transferred 30 μL of the clear supernatant to glass vials with glass inserts. The carotenoids were separated on an Acquity uHPLC system (Waters) fitted with a BEH C18 column (2.1 × 100 mm, 1.7 μm) and a BEH C18 VanGuard pre-column (2.1 × 50 mm, 1.7 μm). Products were separated in a gradient of mobile phase A (50:50 (v/v) methanol/water) and mobile phase B (75:25 (v/v) acetonitrile/ethyl acetate) which were prepared from analytical-grade solvents and passed through a 0.2-μm filter before use. We injected 3 μL of each sample per separation, starting with a ratio of 30% A/70% B for 0.5 min, increasing to 0.1% A/99.9% B for 5.5 min and returning to 30% A/70% B for the final 2 min. The column temperature was maintained at 30 °C and the sample temperature at 8 °C. Continuous online scanning across the UV/visible range (250–600 nm) was carried out using an extended wavelength photo diode array detector (Waters). Carotenoids were quantified from dose–response curves. The separation, detection and quantification of carotenoids, tocopherols, and chlorophylls were carried out as described in detail elsewhere (Fraser et al. 2000).

### Metabolite extraction and GC–MS analysis

Dried seeds were ground to a fine powder using a mortar and pestle, and the dry powder was used for extraction and analysis as previously described (Bligh and Dyer 1959), with modifications. Briefly, 30 mg of the dry powder was mixed with 400 μL of methanol and 400 μL of water and shaken for 1 h in the dark. We then mixed the solution with 800 μL of chloroform and centrifuged at 13,000 × g for 5 min at room temperature to allow phase separation. The epiphase containing polar metabolites was analyzed by gas chromatography mass spectrometry (GC–MS). Another 30 mg of dry powder was saponified with 1 mL 10% (w/v) NaOH for 1 h in a bath sonicator. After centrifugation as above, the supernatant was discarded, and the pellet extracted as described above. The organic phase was analyzed by GC–MS to determine the content of fatty acids, sterols, tocopherols and other isoprenoids.

GC–MS analysis was carried out using 30-µL polar extracts and 600-µL saponified extracts. The samples were transferred to separate glass vials and spiked with corresponding internal standards. Polar samples were spiked with 5 µL 1 mg mL^−1^ ribitol whereas non-polar saponified extracts were spiked with 10 µL 1 mg mL^−1^ deuterated (D27) myristic acid (Cambridge Isotope Laboratories, Cambridge, UK). Samples in glass vials were dried by vacuum centrifugation and derivatized with 30 µL methoxyamine hydrochloride (1 h, 40 °C) and 70 µL MSTFA (2 h, 40 °C) as previously described (Perez-Fons et al. 2014). We then injected 1 µL of the derivatized solution in splitless mode into a 7890B GC on-line with a 5977A mass spectrometer (Agilent Technologies, Palo Alto, CA, USA). Metabolites were separated in a DB-5MS 30 m × 250 μm × 0.25 μm column (J&W Scientific, Folsom, CA, USA) equipped with a 10-m guard column using a temperature gradient of 70–320 °C increasing at 10 °C min^−1^. Helium was used as the carrier gas at a flow rate of 1 mL min^−1^. The inlet was heated to 280 °C and the mass spectrometer transfer line was heated to 250 °C. AMDIS v2.73 was used for peak integration and deconvolution, and to establish author libraries for polar and non-polar metabolites (Perez-Fons et al. 2014). Metabolite levels were normalized against internal standards and corrected by dried weight.

### Starch and soluble sugars

Flag leaves (harvested at 7 pm) and seeds were homogenized under liquid N_2_ and extracted in perchloric acid to measure the starch content, or in ethanol to measure the content of soluble sugars. The quantity of each carbohydrate was determined by spectrophotometry at 620 nm (Juliano 1971; Yoshida et al. 1976). To measure the amylose content, milled rice grains were powdered with a faience pestle and mortar and the powder was transferred to a paper envelope and dried for 1 h at 135 °C. We transferred 100 ± 0.01 mg of dried powder to a conical flask and added 1 mL 95% ethanol and 9 mL 1 M NaOH. The suspension was boiled in a water bath for 10 min, cooled at room temperature for 10 min and then topped up to 100 mL with distilled water. A 5-mL aliquot of the solution was transferred to a 100-mL volumetric flask and mixed with 1 mL 1 M acetic acid, 2 mL 0.2% potassium iodide and 92 mL distilled water. Three amylose solutions (3, 11.5 and 14%) were prepared for comparison. The starch content was determined by measuring the absorbance at 630 nm in a Unicam UV4-100 UV–Vis spectrophotometer after 30 min.

### Microscopy

For light microscopy, seed chips (~ 1 mm^3^) were fixed in 2.5% (v/v) glutaraldehyde in 0.1 M sodium phosphate buffer (pH 7.2) overnight at 4 °C and washed three times with the same buffer. Semi-thin Sects. (2 µm) were prepared using an Ultracut E Ultra Microtome 701,704 (Reichert Technologies, Buffalo, NY, USA). The sections were transferred to microscope slides and stained with Richardson’s blue before adding a drop of DPX mounting medium and a coverslip. The slides were observed under a DM4000 B microscope (Leica Microsystems, Wetzlar, Germany) and photographed using a Leica DFC300 FX 1.4-megapixel digital color camera equipped with the Leica software application suite LAS v3.8.

For transmission electron microscopy (TEM), seeds fixed as above were post-fixed in 1% (w/v) osmium tetroxide in 0.1 M sodium phosphate buffer (pH 7.2) for 1 h, washed three times in redistilled water and dehydrated in an alcohol series (30–100%) before embedding in epoxy resin (Electron Microscopy Sciences, Hatfield, PA, USA) and polymerizing at 60 °C for 48 h. Ultra-thin Sects. (70–90 nm) were prepared using the Ultracut E Ultra Microtome and were mounted on Formvar carbon-coated copper grids. The sections were stained with uranyl acetate (10 min) and Reynold’s lead citrate (2 min) prior to examination using an EM 910 device (Carl Zeiss, Oberkochen, Germany).

### Statistical analysis

Data were analyzed for statistical significance using JMP Pro (SAS Institute, Cary, NC, USA), *R* (www.r-project.org), and Mathematica (Wolfram Research, Champaign, IL, USA). Linear models were used to determine statistically significant dependencies and differences in normalized gene expression analysis, carbohydrate analysis, GC–MS analysis and phytohormone analysis. Significant differences were inferred using Student’s t-test (*P < 0.05, **P < 0.001) (JMP Pro) or by calculating 95% confidence intervals (*R* and Mathematica). We accepted only those results that were statistically significant in all three platforms.

### Network analysis

Network calculations were carried out in Mathematica, with the abundance of each transcript renormalized to *OsActin1* in wild-type rice. Changes in RNA abundance relative to wild-type plants were pooled over all lines. Quantiles 0.025 and 0.975 of the pooled set of changes were taken as the threshold for statistical significance (*p* < 0.05). Using the data for the normalized absolute RNA abundances for individual experiments, we created linear models of the changes in native gene expression as a function of the abundance of each heterologous mRNA. We calculated the adjusted coefficient of determination (AR^2^), the strength of the effect (α), and the *p*-value of that strength for each model. We accepted only those results for which AR^2^ > 0.2, |α|> 0.2 and *p* < 0.05. Hormone levels were also correlated to macroscopic plant phenotypes, gene expression in seeds, and metabolite levels by calculating Spearman correlations.

## Results

### Recovery and characterization of plants expressing ectopic MVA pathway genes

Transformation experiments were carried out to introduce a complete ectopic MVA pathway into rice, followed by endosperm-specific transgene expression and the plastidial import of the corresponding enzymes (Fig. 1). Plants were transformed with all five genes of the MVA pathway on two separate cassettes (*BjHMGS:tHMGR:CrMK* + *CrPMK:CrMVD*). *OsWR1* was included in the transformation cocktail in separate experiments. Transgenic plants without WR1 only survived for ~ 1 week after transfer to soil, whereas those with WR1 were viable.

**Fig. 2:**
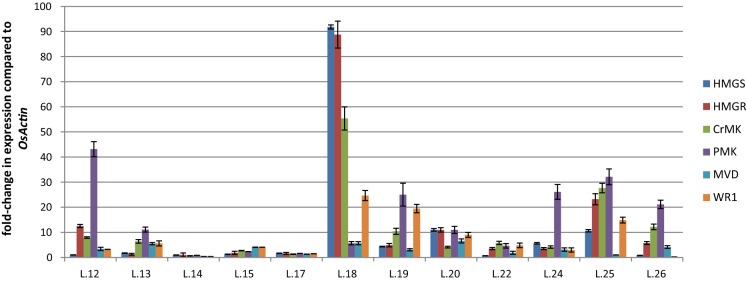
Expression profiles of *BjHMGS*, *tHMGR*, *CrMK*, *CrPMK*, *CrMVD* and *OsWR1* normalized to *OsActin* in 12 transgenic lines with the highest expression levels in T2 seeds

We recovered ~ 50 independent transformants carrying all MVA + WR1 input transgenes and identified at least 20 lines expressing the transgenes in T1 seeds. The seeds were germinated to produce T1 plants, which were selfed to produce T2 seeds. We selected the 12 lines with the highest levels of transgene expression in T2 seeds (normalized to the expression of endogenous *OsActin1*) for further in-depth analysis (Fig. 2). Compared to *OsActin1*, the ranges of transgene expression were 0.8–91-fold (*BjHMGS*), 1–88-fold (*tHMGR*), 0.5–55-fold (*CrMK*), 0.88–43-fold (*CrPMK*), 0.4–6.5-fold (*CrMVD*), and 0.2–24-fold (*OsWR1*).

### Phenotypic analysis

The ability of the transgenic lines to produce T2 seeds confirmed that the expression of ectopic MVA enzymes did not affect fertility. We also compared the 12 transgenic lines to 12 wild-type plants in order to determine whether the ectopic pathway affected developmental traits such as height, leaf number, leaf morphology, leaf chlorophyll content, and senescence. The MVA + WR1 lines were shorter than wild-type plants (1.4 to twofold) in 11/12 lines (line 22 had similar height to wild type); with fewer leaves (1.25–1.9-fold) in 11/12 lines (line 22 had the same number of leaves as the wild type) and a smaller leaf area in all 12 lines (1.9–4.9-fold) (Supplementary Figure S1 a,b). Furthermore, the transgenic lines entered senescence prematurely (12 weeks) compared to wild-type plants (20 weeks) and their leaves accumulated less chlorophyll (1.3–1.5-fold) in 10/12 lines (Lines 13 and 20 had similar levels to wild-type). The differences in height, leaf area, leaf chlorophyll content and onset of senescence between MVA + WR1 lines and wild-type plants were statistically significant (Supplementary Figure S1 c).

### Analysis of phytohormone levels in leaves

**Fig. 3:**
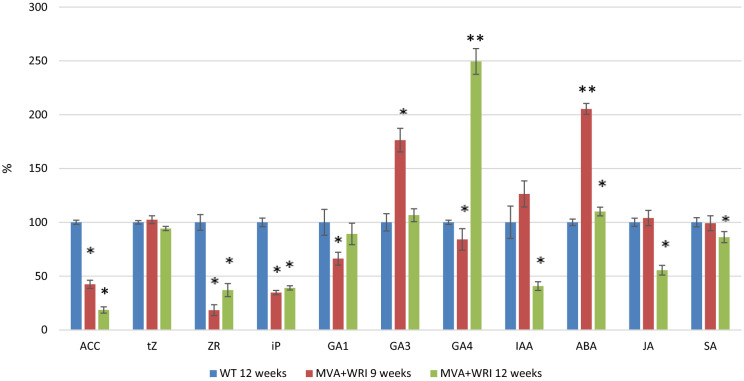
Phytohormone content of MVA + WR1 plants after 9 and 12 weeks growing in soil, relative to wild-type controls set at 100%. The 9 and 12 week time points represent times before and after the onset of senescence. Asterisks indicate a statistically significant difference between wild-type (WT) and transgenic lines. Values are means ± SD (n = 3 biological replicates) as determined by Student’s t-test (*P < 0.05). ACC = 1-aminocyclopropane-1-carboxylic acid, tZ = *trans*-zeatin, *ZR*  zeatin riboside, *iP*  isopentenyl adenine, *GA1/GA3/GA4*  gibberellins, *IAA*  indole-3-acetic acid, *ABA*  abscisic acid, *JA*  jasmonic acid, *SA*  salicylic acid

To understand how an endosperm-specific pathway could induce such pleotropic changes, we hypothesized that additional flux through the MVA pathway altered phytohormone levels generating phenotypes similar to those reported following the disruption of phytohormone synthesis or homeostasis (Outlaw 2003; Yuan et al. 2010; Kumar et al. 2018). This could reflect retrograde signaling from the plastids and mitochondria to indicate their physiological and developmental status to the nucleus, allowing the modulation of gene expression by phytohormones (Kleine and Leister, 2016). We therefore measured the levels of ABA, isopentenyl adenine, jasmonic acid, ACC, *trans*-zeatin, zeatin riboside, IAA, salicylic acid and three forms of gibberellic acid (GA1, GA3 and GA4) in the leaves of all MVA + WR1 lines and wild-type controls after 9 and 12 weeks growing in soil, representing time points before and around the onset of senescence in these lines (Fig. 3).

There was a major and significant decrease in the levels of zeatin riboside (3- to fivefold at 9 weeks) in all the lines. At 12 weeks, zeatin riboside levels were still lower that the wild type (1.5 to 2.7-fold) in 11 of the 12 lines (line 18 was indistinguishable from wild type). Isopentenyl adenine levels also decreased significantly in 11/12 lines (2.9 to eightfold) at 9 weeks (line 15 had very similar levels to wild-type). At 12 weeks all 12 lines contained 1.5 to fourfold lower levels compared to wild type. ACC levels were lower at 9 (1.3–4.9-fold) and 12 (5.4–9.8-fold) weeks in 11/12 lines (Line 25 had similar levels to wild type). Less striking but still significant decreases in the levels of IAA (1.27 to 2.45-fold) were measured in 10/12 lines after 12 weeks in soil (lines 25 and 26 contained similar levels compared to wild-type). At 9 weeks, levels of ABA were significantly higher in 9/12 transgenic lines at 9 weeks (1.3 to 3.6-fold) with lines 12, 13 and 14 showing levels similar to wild-type. At 12 weeks, however, ABA levels were similar in 11/12 transgenic lines with wild-type plants, with only line 24 exhibiting an increase (1.56-fold). Jasmonic acid levels were similar in wild type and in 11/12 transgenic lines at 9 weeks, with only line 26 showing an increase (1.6-fold), but dropped significantly in 11/12 lines after 12 weeks (1.4–4.8-fold) (line 17 contained similar levels compared to wild-type). The levels of *trans*-zeatin appeared to be consistent at all time points in all plants whereas zeatin riboside was, as stated above, much more abundant in the wild-type plants than any of the transgenic lines. ACC was more abundant in wild-type plants than MVA + WR1 plants at both time points, but the level of this hormone declined in the transgenic plants between 9 and 12 weeks in all 12 lines we analyzed. GA3 was more abundant in 9/12 lines compared to wild-type plants at 9 weeks (2.01 to 10.7-fold). Lines 17, 18 and 19 contained similar GA3 levels compared to wild-type) but fell back to basal levels (same as wild type) by 12 weeks in 10/12 lines (lines 15 and 22 had an increase of 1.75-fold); GA1 dipped significantly below wild-type levels at 9 weeks in 10/12 lines (1.3–4.3-fold). The remaining 2 lines (lines 14 and 20) had an increase of 1.7 fold, but recovered to normal levels (as in the wild type) by 12 weeks in 10/12 lines (lines 20 and 24 showed a twofold decrease). In contrast, GA4 was depleted in 10/12 transgenic lines at 9 weeks (lines 17 and 22 showed an increase of 4.5-fold), but at 12 weeks, 10/12 lines had recovered to basal levels (same as wild type) and lines 18 and 24 had an increase of 2.1 fold).

In summary, the levels of most phytohormones at 12 weeks were significantly lower in MVA + WR1 plants compared to wild-type plants, with the exception of GA4 and ABA. There was a significant positive correlation between the levels of some phytohormones, although tZ and ACC showed a clear negative correlation (Supplementary Table 4).

### Network analysis of native MVA and MEP pathway gene expression

**Fig. 4:**
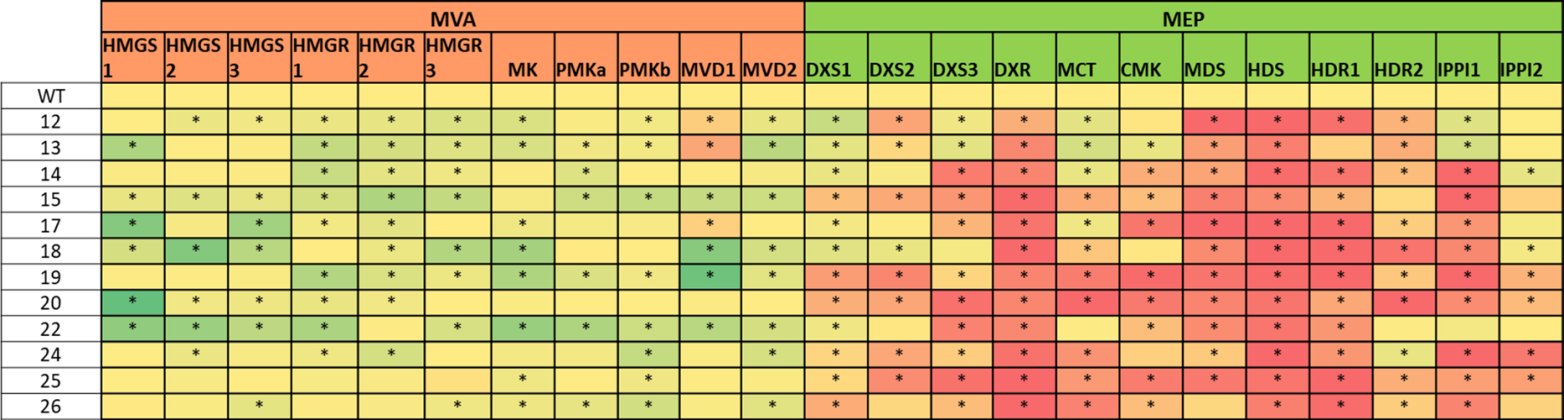
Heat map showing fold-changes in the expression of endogenous MVA and MEP pathway genes in T2 seeds when comparing wild-type and transgenic plants (left column) expressing an ectopic MVA pathway. The red gradient shows increasing degrees of downregulation and the green gradient shows increasing degrees of upregulation, with yellow indicating no change in expression (onefold). The red gradient is expanded in the lower ranges because this is where most of the values lie, whereas the green gradient is linear. Asterisks indicate a statistically significant difference between wild-type (WT) and transgenic lines. Values are means ± SD (n = 3 technical replicates) as determined by Student’s t-test (*P < 0.05)

Given the extensive feedback regulation of the MVA and MEP pathways, we wanted to investigate interactions between ectopic and native gene expression in the MVA + WR1 lines. We considered the expression levels of 13 endogenous genes representing the native MVA pathway (*OsHMGS, OsHMGR, OsMK, OsPMK, OsMVD*) and MEP pathway (*OsDXS, OsDXR, OsMCT, OsCMK, OsMDS, OsHDS, OsHDR* and *OsIPPI*), focusing on the dependencies between the various MVA/MEP genes. We constructed a heat map of the expression profiles based on percentiles to visualize the most significant changes compared to wild-type plants. All MVA + WR1 lines showed a small but statistically significant upregulation of the endogenous MVA pathway and a much stronger statistically significant downregulation of the MEP pathway (Fig. 4). The endogenous genes *OsDXR* and *OsHDS* were significantly modulated in all 12 lines, whereas *OsMDS* and *OsHDR1* were significantly downregulated in > 90% of the lines, *OsIPPI1* was significantly downregulated in > 75% of the lines and *OsDXS2, OsDXS3, OsCMK* and *OsMCT* were significantly downregulated in > 50% of the lines.

**Fig. 5:**
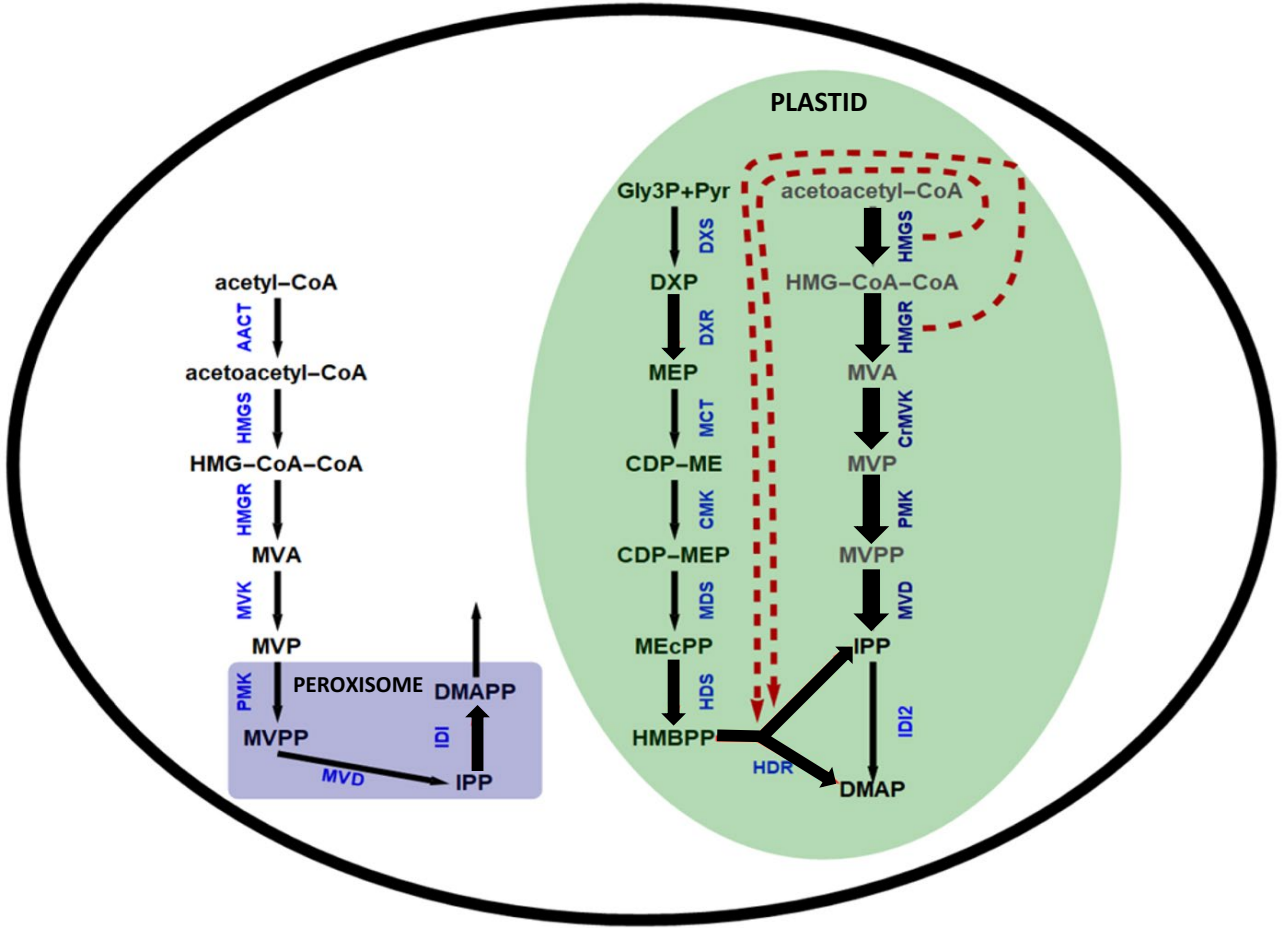
Network analysis of MVA + WR1 lines. Small arrows: endogenous pathways. Medium arrows: endogenous genes significantly downregulated in more than 50% of the transgenic lines compared to wild-type plants. Big arrows: ectopic MVA pathway. Dashed arrows indicate a significant (*p* < 0.05) and strong positive correlation (α < –0.2) between absolute transcript levels of the transgene from which the arrow points and absolute transcript levels of the endogenous genes to which the arrow points

To gain a better understanding of the interactions between ectopic and endogenous gene expression and to identify compensatory expression between functionally orthologous genes, we performed a network analysis of changes in expression. This showed that the early genes of the endogenous MVA pathway (*OsHMGS* and *OsHMGR*) were upregulated by up to five-fold whereas those at the beginning and end of the endogenous MEP pathway (*OsDXR, OsMDS, OsHDS, OsHDR1* and *OsIPPI1*) were downregulated by more than two-fold compared to wild-type plants (Figs. 4 and 5).

There was a negative correlation between two transgenes (*BjHMGS* and *tHMGR*) and the endogenous gene *OsHDR*. We also observed a strong positive correlation (|α|> 0.2) between two transgenes (*OsWR1* and *CrMK*) and transgene OsWR1 with the endogenous gene *OsMK1* albeit with a borderline significance value of *p* < 0.06 (Fig. 5). The correlations between transgene and endogenous gene expression in the network analysis of each line are summarized in Supplementary Figure S2.

### Metabolomic analysis

To understand how gene expression changes propagated to the metabolic level, we measured the levels of isoprenoid-related metabolites such as pheophytin, lutein and α-tocopherol by HPLC analysis (Supplementary Figure S3 a). Although pheophytin is a chlorophyll degradation product and there is no chlorophyll in mature rice seeds, the developing green seeds do contain chlorophyll, explaining the origin of this product. All MVA + WR1 lines accumulated more α-tocopherol than wild-type plants (mean = 7.51 mg g^−1^ dry weight compared to 4.3 mg g^−1^ in wild-type plants, representing a 1.75-fold increase in the transgenic lines). Furthermore, one third of the lines accumulated lutein and pheophytin (mean values of 1.88 and 3.9 mg g^−1^ dry weight, respectively) which were not detected in the wild-type seeds.

We also measured the content of intermediate metabolites by GC‐MS (Supplementary Figure S3 b-d), revealing that 100% of the lines accumulated more fatty acids than the wild-type controls (minimum 1.54-fold and maximum 6.22-fold difference). We also found that 58% of the lines accumulated more isoprenoids than the wild-type plants (minimum 1.17-fold and maximum 2.9-fold difference). Another 34% of the plants showed no significant change in isoprenoid levels. The analysis of squalene levels revealed a decrease (minimum 1.04-fold and maximum 3.47-fold difference) with respect to wild-type levels in 83% of the lines and an increase in 17% of the lines. Other sterols also accumulated to higher levels compared to wild-type plants (Supplementary Figure S4). Specifically, campesterol levels increased by 1.41–2.24-fold in 25% of the lines, stigmasterol levels increased by 1.53–3.56-fold in 50% of the lines, β-sitosterol levels increased 1.32–2.92-fold in 58% of the lines, and cycloartenol levels increased 1.91–7.20-fold in 58% of the lines.

### Quantitation of starch and soluble sugars

Given the observed changes in fatty acid levels, we also investigated the effect of the ectopic MVA pathway on primary carbohydrate metabolism by measuring the levels of starch and soluble sugars in T1 leaves and T2 seeds (Supplementary Figure S5). In the leaves, we observed a 2.59-fold decrease in starch levels compared to wild-type, with a concomitant 2.73-fold increase in the content of soluble sugars. In contrast, we observed the opposite profile in the seeds. The MVA + WR1 lines showed a 1.78-fold increase in the seed starch content and a smaller 1.31-fold compensatory reduction in soluble sugar levels. A heat map showing the changes in starch and sugar levels relative to wild-type plants in all 12 lines is provided in Supplementary Figure S6.

### Seed morphology

**Fig. 6:**
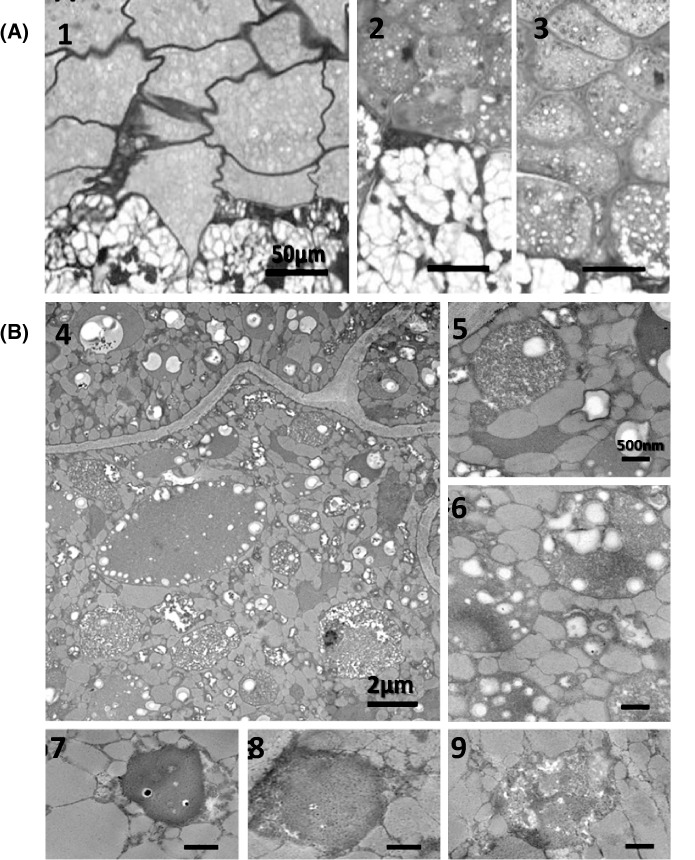
(**a**) Light microscopy of rice seeds. (1a) Aleurone layer and starch grains of wild-type seed. (2a) Aleurone layer and starch grains of line 19 expressing MVA-WR1. (3a) Aleurone layer and starch grains of line 24 expressing MVA-WR1. Scale bar = 50 µm. (**b**) Transmission electron microscopy of wild-type and transgenic seeds. (4-6b) Wild-type seed showing diverse types of plastids and starch granules. Scale bar = 2 µm (4) and 500 nm (5, 6). (7, 8b) Plastids in transgenic line 19, showing small black spots (fatty acid accumulation). (9b) Plastid in transgenic line 18, showing a deformed and unstructured membrane. Scale bar = 500 nm (d-f)

Finally, we addressed the impact of metabolic changes on cell and plastid morphology. We compared the morphology of wild-type seeds to T2 transgenic seeds from line 19, which was selected because the transgene expression levels were mid-range values among the 12 candidate lines. We observed no differences in the aleurone layer, but striking differences in the endosperm. Starch granules were much more abundant in the seeds from line 19 compared to wild-type controls (Fig. 6a).

We investigated the ultrastructure of the plastids in the endosperm by TEM, revealing distinct morphological changes in MVA + WR1 lines 18 and 19 (Fig. 6b). The outer plastid membrane is usually rounded, but this was not the case in the transgenic lines. Although similar to wild-type plastids in gross structure, we observed small black spots representing fatty acid deposits and an imperfectly rounded membrane in the transgenic lines.

## Discussion

### Rationale and generation of transgenic plants

All isoprenoids are derived from the precursors IPP and DMAPP via the MVA or MEP pathways. Most organisms only possess one of these pathways, but plants have both and each pathway can be modified by metabolic engineering to alter the isoprenoid profile qualitatively or quantitatively (Shi et al. 2014). The metabolic engineering of isoprenoids has been carried out mainly in microbes such as the bacterium *Escherichia coli* or the yeast *S. cerevisiae* (Ward et al. 2018) because they are simpler than higher plants (Zhu et al. 2013; Farré et al. 2014). These studies have involved the expression of heterologous genes encoding HMGR, HMGS or DXR and aimed to overcome the strict regulation of flux (Singh et al. 2014; Wang et al. 2012; Shi et al. 2014). Typically, the studies focused on the accumulation of a particular target compound but neglected the wider metabolic impact of the ectopic enzymes (Jayashree et al. 2018; Wang et al. 2011). We hypothesized that the strict regulation of the cytosolic MVA pathway in plants could be overcome by introducing an ectopic MVA pathway in the plastids, thus increasing the production of IPP and DMAPP. However, the increase in precursor availability might trigger pleiotropic effects given that many phytohormones are isoprenoid derivatives. In turn, phytohormones can influence the metabolic activity of plants, so the ultimate steady state generated by the increase in flux through the MVA pathway is difficult to predict.

To investigate the effects in more detail, we introduced two versions of the ectopic MVA pathway into rice endosperm, one consisting of all five MVA pathway enzymes (BjHMGS, tHMGR, CrMVK, CrPMK and CrMVD) and the second comprising the same five enzymes plus the transcriptional regulator OsWR1. All five enzymes were endowed with a plastid targeting peptide because direct plastid transformation is not yet possible in monocots, whereas OsWR1 was directed to the nucleus by an intrinsic NLS. We expressed a truncated version of HMGR (*tHMGR*) because the endogenous version is strictly regulated at multiple levels (Chappell et al. 1995) whereas the truncated version is uncoupled from much of this regulatory burden (Hemmerlin 2013). We expressed the five-gene MVA pathway to provide a full complement of enzymes in the plastid, but none of the corresponding plants survived more than a week after transfer to soil. Given the limited pool of the precursor acetyl-CoA available in the plastids, we hypothesized that the plants suffered an acetyl-CoA deficit and thus added the WR1 transcription factor to increase the availability of acetyl-CoA precursors (Cernac and Benning 2004). We regenerated ~ 50 of the MVA + WR1 transformants and selected 12 lines with the highest transgene expression levels in T2 seeds. We investigated the macroscopic phenotype (including seed morphology) and measured the metabolites derived from IPP and DMAPP, transgene and endogenous MVA/MEP pathway gene expression in T2 seeds compared to the *OsActin1* gene, and the levels of various phytohormones, carbohydrates and fatty acids.

### Ectopic gene expression and its relationship with phenotype and phytohormone levels

Blocking isoprenoid synthesis is generally deleterious but the overexpression of MVA pathway enzymes is not usually associated with adverse morphological phenotypes. However, we found that our MVA + WR1 lines were shorter than wild-type plants, had smaller leaves (with lower chlorophyll levels), and entered senescence early. The observation of these distinct phenotypes led us to hypothesize that the transgenes affected phytohormone production.

Network analysis did not indicate a significant relationship between transgene expression and hormones, although GA4 levels explained 31% of the variation in plant height. Gibberellins are derived from the MEP pathway and we found that GA1 and GA4 were depleted in transgenic lines at 9 weeks, commensurate with the suppression of the MEP pathway genes, but accumulated again by 12 weeks thus indicating changes taking place after the onset of senescence. This may reflect the positive regulation of gibberellin synthesis in rice by brassinosteroids, which are derived from the endogenous MVA pathway (Tong et al. 2014). ABA and lutein are the end points of the two branches of the carotenoid pathway, and we observed corresponding increases in both metabolites in transgenic lines. Jasmonic acid was less abundant in the transgenic lines than wild-type plants. Similarly, the ethylene precursor ACC was depleted in transgenic lines, and this may reflect an increase in the abundance of amino acids, which can be converted into metabolites that inhibit ACC production (Taiz et al. 2015). Acetyl-CoA can be redirected to other pathways including the synthesis of sugars, fatty acids and amino acids (Hemmerlin et al. 2012). Although we did not directly measure the levels of free amino acids in our transgenic lines, the observed increase in sugars and fatty acids is consistent with our hypothesis.

### Modulation of ectopic MVA and endogenous MVA/MEP gene expression

The endogenous MVA and MEP pathways are regulated at multiple levels, including the level of transcription. We therefore compared the transgenic and wild-type plants for the expression of 13 endogenous genes in the MVA pathway and 12 in the MEP pathway, representing multiple paralogs of HMGS, HMGR, MVD, DXS, HDR and IPPI, alternative splice variants of PMK, and single-copy genes encoding MK, DXR, MCT, CMK, MDS and HDS. We anticipated an increase in endogenous MVA gene expression reflecting cross-talk with the ectopic pathway and partial suppression of the MEP pathway due to feedback inhibition caused by the accumulation of excess IPP and DMAPP in the plastids. Broadly, our experimental data matched these predictions. We observed a general increase in the expression of endogenous MVA pathway genes and the suppression of MEP pathway genes. Specifically, the MVA pathway genes encoding MK and multiple isoforms of HMGS and HMGR were induced, whereas the MEP pathway genes encoding DXR, MDS and HDS were strongly repressed. Although our study is the first to report the effect of an ectopic MVA pathway in cereals (and in the seeds of any plant species), earlier work in tomato (*Solanum lycopersicum*) plants revealed that the overexpression of HMGS in the cytosol triggered the upregulation of the endogenous MVA pathway gene *LeHMGR1* and led to the increased production of squalene, carotenoids and sterols (Liao et al. 2018). Interestingly, the authors also reported the upregulation of endogenous MEP pathway genes (*LeDXS1*, *LeDXS2* and *LeDXR*) in contrast to our results. Intriguingly, we found that the two rice paralogs of IPPI showed distinct responses to the ectopic MVA pathway, with *IPPI1* strongly repressed and *IPPI2* only weakly repressed. This may reflect the toxicity of IPP/DMAPP (Sivy et al. 2011), which may drive their export from the plastids (Wang et al. 2019) given that the natural function of IPPI is to dynamically adjust the IPP/DMAPP ratio (Tritsch et al. 2010). IPPI1 may be suppressed more strongly than IPPI2 because the former is expressed in more tissues and its activity would therefore lead to more widespread DMAPP toxicity. In contrast, IPPI2 is the only isoform expressed in plastids (Jin et al. 2019), where the MEP pathway is downregulated in our transgenic lines. This would limit the size of the DMAPP pool and release the enzyme from suppression.

Network analysis can provide insight into the relationship between transgene expression and endogenous responses in plants at the levels of the transcriptome and metabolome, as previously reported for maize (Farré et al. 2013), *A. thaliana* and rice (Movahedi et al. 2011). Not only did we observe general enhancement of the endogenous MVA pathway and general suppression of the endogenous MEP pathway, but also specific positive correlations between single transgenes (or transgene pairs) and individual endogenous genes, specifically a negative correlation between transgenes *BjHMGS* + *tHMGR* and the endogenous gene *OsHDR* in MVA + WR1 lines.

### Endogenous secondary metabolism is modulated by the ectopic MVA pathway

The overexpression of individual MVA genes in plastids has been shown to cause a general increase in isoprenoid levels by eliminating metabolic bottlenecks, but the impact at the level of specific classes of isoprenoids is more complex. For example, Enfissi et al. (2005) expressed HMGR in tomato plastids and observed a 2.4-fold increase in total phytosterol levels with little or no impact on the carotenoid content, whereas Liao et al. (2018) expressed HMGS in tomato plastids, which increased squalene levels by 1.4-fold, sterol levels by ~ fivefold, α-tocopherol levels by ~ fivefold, lycopene levels by ~ twofold, and β-carotene levels by ~ 2.5-fold. Furthermore, Kumar et al. (2012) expressed a complete ectopic MVA pathway in tobacco plastids and reported a 1.2-fold increase in fatty acid levels, a tenfold increase in squalene levels and a 1.4-fold increase in β-carotene levels. The advantages of rice endosperm as a metabolically inactive chassis allowed us to perform a much more comprehensive analysis of metabolic products and intermediates in our transgenic lines without interference from the metabolic housekeeping functions of tissues such as leaves and fruits. We found that certain classes of isoprenoid levels increased in most of our MVA + WR1 lines. In terms of specific compound classes, targeted metabolomics analysis revealed that (1) α-tocopherol levels increased in all our transgenic lines (mean = 1.75-fold); (2) the levels of most sterols also increased, but exceptionally squalene levels decreased in 83% of the transgenic lines; and (3) carotenoids accumulated in the endosperm of the transgenic lines even though endogenous carotenoid synthesis is blocked due to the very low expression of the first enzyme in the pathway, phytoene synthase (Bai et al. 2016).

Tocopherols are synthesized via three routes: directly from the MEP pathway (phytyl-2-phosphate), via erythrose-4-phosphate and phosphoenolpyruvate converging on the shikimic acid pathway, and via pheophytin from the chlorophyll degradation pathway (Almeida et al. 2011). Given that the ectopic MVA pathway was shown to suppress the endogenous MEP pathway, we speculate that the excess tocopherols did not arise directly from the MEP pathway and were instead derived indirectly via glycolysis or chlorophyll degradation in the immature green seed. In the glycolysis scenario, MVA pathway intermediates could overspill into the production of acetyl-CoA and pyruvate, which feed into glycolysis/pentose phosphate, amino acid and fatty acid metabolism (Hemmerlin et al. 2012). In agreement with this, we detected a strong increase in the accumulation of fatty acids, indicating that the expression of OsWR1 enhanced the acetyl-CoA pool and thus promoted tocopherol biosynthesis indirectly. In the chlorophyll degradation scenario, tocopherol is derived from the breakdown of chlorophyll in green rice seeds (Jalink et al. 1999). This would be the most likely route in the lines that contained not only tocopherol but also a mix of pheophytin and lutein.

Squalene is a sterol produced directly downstream from the MVA pathway (Vranova et al. 2013). As stated above, the overexpression of endogenous MVA pathway genes in the transgenic lines may promote IPP export from the plastids to the cytosol, hence we expected to see higher levels of squalene in our plants. Instead we observed lower levels, which may reflect the conversion of squalene into downstream products such as campesterol, stigmasterol, β-sitosterol, sitosterol, cycloartenol, cholesterol, and/or brassinosteroids (Holmberg et al. 2002). We detected higher levels of campesterol, stigmasterol, β-sitosterol and cycloartenol in a proportion of the transgenic lines (ranging from 25% in the case of campesterol and 50% in the case of stigmasterol to 58% in the case of β-sitosterol and cycloartenol) and the magnitude of the increase ranged from 1.32-fold to 7.20-fold. Furthermore, we indirectly predicted an increase in brassinosteroid levels due to the increase in gibberellin levels from the downregulated MEP pathway.

The presence of carotenoids in the endosperm of our transgenic rice lines may reflect the activation of the *PSY1* gene (encoding phytoene synthase) by the higher levels of IPP/DMAPP. Alternatively, the cytochrome P450 monooxygenase OsCYP97A4 was previously shown to promote the accumulation of lutein in rice plants because it can add hydroxyl groups to both β-rings of β-carotene (Lv et al. 2012). It is possible that *OsCYP97A4* is expressed at higher levels in our transgenic lines or that the protein is more stable, enabling it to accumulate. Increasing the availability of IPP appears to boost flux through the carotenoid pathway, which is regulated in a complex manner (Bouvier et al. 2005; Botella-Pavia et al. 2004; Fraser et al. 2009).

### Adjustment of endogenous primary metabolism

The synthesis of isoprenoids requires energy and precursors from primary metabolism, and in the transgenic lines this is likely to be sourced from the starch reserves of the endosperm by converting the starch into soluble sugars and then into precursors such as acetyl-CoA and pyruvate if no alternative source is available, as previously reported in maize (Decourcelle et al. 2015). We therefore anticipated the MVA + WR1 lines would potentially store more starch and release less sugar into the endosperm because additional energy is provided independently by using WR1 to boost the levels of acetyl-CoA. We did not anticipate any significant changes in the leaves.

Our results in the seeds were consistent with these observations. The MVA + WR1 lines produced excess acetyl-CoA, which was converted into starch for storage. Although the seeds behaved as anticipated, we also observed changes in the leaves. The starch reserves in the leaves were depleted and soluble sugars accumulated. Metabolic interventions affecting endosperm starch reserves have previously been shown to affect the starch/sugar balance in rice leaves. For example, the *OsAPL2* gene encodes an enzyme in the starch biosynthesis pathway expressed solely in the endosperm, yet targeted mutations in this gene also depleted starch reserves in the leaves and increased the soluble sugar content (Perez et al. 2018). These results suggest that the ectopic MVA pathway affects the source–sink balance during seed development by utilizing starch and has a knock-on effect on primary starch biosynthesis in the leaves. Alternatively, the observed effects may reflect the changes in phytohormone levels discussed above, which are also known to affect rice seed development. For example, the treatment of rice with an inhibitor of ABA biosynthesis (nordihydroguaiaretic acid) did not affect ABA but reduced the levels of jasmonic acid and increased the grain yield, whereas an inhibitor of ethylene biosynthesis (2-aminoisobutyric acid) boosted photosynthesis in the flag leaves and increased the starch content of the leaves (Tamaki et al. 2015). The lower levels of ACC and jasmonic acid in our MVA + WR1 lines thus correlated with the increase in seed starch.

Given that starch accounts for ~ 80% of the total dry matter of the rice endosperm, we anticipated that the changes in starch levels in the seeds would affect seed morphology and the ultrastructure of the plastids. Light microscopy revealed no changes to the aleurone layer but significant changes in the endosperm of the MVA + WR1 seeds, with a much larger number of starch grains commensurate with the modulation of starch levels. Likewise, the plastids of the MVA + WR1 lines featured black spots indicating fatty acid deposits, and although the general morphology was similar to plastids in wild-type plants the membrane was less well structured. Plant cells have delicate response mechanisms that sense fatty acid levels and adjust metabolism accordingly (Eccleston and Ohlrogge 1998). In leaves, excess fatty acids are degraded by β-oxidation in peroxisomes. The higher level of triacylglycerols in the MVA + WR1 lines reflects the interplay between the enhanced availability of isoprenoid substrates in the plastids, the effects of isoprenoid intermediates on fatty acid synthesis (Kizer et al. 2008), and the balance between more active fatty acid synthesis in the plastid and the degradation of excess fatty acids in the cytosol. The relationship between fatty acid content and plastid structure has been reported in *A. thaliana* plants overexpressing *FATTY ACID ELONGATION 1*, in which the accumulation of long-chain fatty acids caused a dose-dependent effect on the structure and ultrastructure of the plastid membrane (Millar et al. 1998).

## Conclusions

The coexistence of two different pathways (ectopic MVA and native MEP pathways) in the plastids of rice endosperm provided an unprecedented opportunity to study the regulation of isoprenoid biosynthesis in a tissue that is almost metabolically inactive. Transgenic plants lacking OsWR1 only survived ~ 1 week after transfer to soil because acetyl-coA was not available for essential pathways. Ectopic MVA + WR1 expression in plastids produced a strong downregulation of endogenous MEP pathway. The ectopic MVA + WR1 pathway enhanced isoprenoid biosynthesis by generating surplus IPP/DMAPP, which we confirmed indirectly by measuring a general increase in the levels of sterols and tocopherols, as well as other isoprenoids such as squalene and lutein. Expression of all five MVA pathway genes plus OsWR1 had a negative impact on growth leading to stunting and underwent early senescence (reflecting the change in phytohormone levels) and a decrease in chlorophyll content but there was no depletion of seed starch because the *OsWR1* gene ensured that sufficient acetyl-CoA was made available. This increased fatty acid levels by up to 6.22-fold, causing the excess to accumulate in the plastids and influence the structure of the membrane. Intriguingly, the transgenic endosperm tissue was also able to produce carotenoids even though this pathway is normally blocked by the rate-limiting activity of the enzyme phytoene synthase.

The analysis of gene expression, primary and secondary metabolites, and phytohormones produced a highly complex network of interactions that was difficult to decipher, making it almost impossible to predict the impact of specific metabolic interventions. Changes in the isoprenoid pathway can affect the synthesis of multiple phytohormones, and the crosstalk between phytohormone signaling pathways can influence the regulation of metabolism at the transcriptional and post-transcriptional levels (Ohri et al. 2015). It is challenging to monitor the levels of all pathway intermediates, particularly transient precursors such as the key molecules IPP and DMAPP.

## Supplementary Information

Below is the link to the electronic supplementary material.

Supplementary file1 (DOCX 1608 kb)
