# Supplementary material for: Multilevel interactions between native and ectopic isoprenoid pathways affect global metabolism in rice
Source: Transgenic Res. 2022 Feb 24;31(2):249–68. doi: 10.1007/s11248-022-00299-6 (PMC8993735; doi:10.1007/s11248-022-00299-6)
Supplement: Supplementary file 1 — Supplementary file1 (DOCX 1608 kb) [file 11248_2022_299_MOESM1_ESM.docx]

**Title:** Multilevel interactions between native and ectopic isoprenoid pathways affect global metabolism in rice.

**Author names and affiliations:** Lucía Pérez^1^, Rui Alves^2^, Laura Perez-Fons^3^, Alfonso Albacete^4,5^, Gemma Farré ^1^, Erika Soto^6^, Ester Vilaprinyó^2,7^, Cristina Martínez-Andújar^4^, Oriol Basallo^2^, Paul D. Fraser^3^, Vicente Medina^1^, Changfu Zhu^1^, Teresa Capell^1^, Paul Christou^1,8^*

^1^Department of Plant Production and Forestry Science, School of Agrifood and Forestry Science and Engineering (ETSEA), University of Lleida-Agrotecnio Center, Lleida, Spain

^2^Departament de Cienciès Mèdiques Bàsiques, Universitat de Lleida, Lleida, Spain

^3^ School of Biological Sciences, Royal Holloway University of London, Egham Hill, UK

^4^ Departament of Plant Nutrition, Center of Edaphology and Applied Biology of the Segura (CEBAS), Consejo Superior de Investigaciones Científicas (CSIC), Campus Universitario de Espinardo, E-30100, Espinardo, Murcia, Spain

^5^ Department of Plant Production and Agrotechnology, Institute for Agri-Food Research and Development of Murcia, La Alberca, Murcia, Spain

^6^Department of Chemistry, University of Lleida-Agrotecnio Center, Lleida, Spain

^7^ IRBLleida, Lleida, Catalunya, Spain

^8^Catalan Institute for Research and Advanced Studies (ICREA), Barcelona, Spain

***Author for correspondence**: Paul Christou, Department of Plant Production and Forestry Science, School of Agrifood and Forestry Science and Engineering (ETSEA), University of Lleida-Agrotecnio Center, Av. Alcalde Rovira Roure 191, 25198 Lleida, Spain.

Tel: (+34)973702693, [paul.christou@udl.cat](mailto:paul.christou@udl.cat)

**Supplementary Tables**

| **Gene** | **Forward Sequence** | **Reverse sequence** |
| --- | --- | --- |
| **BjHMGS** | 5′- CATCTACTTCCCGCCAACCTG 3′ | 5′- TAGCCGCTTCATCAATGCTGG-3′ |
| **AtHMGR** | 5′- GGTTGCGTCCACGAATAGAGG-3′ | 5′- TTCCCTGCGTGAGCGTTGA-3′ |
| **CrMK** | 5′- CCTTTTGCGTGGCTTTGTCAG -3′ | 5′- AAACGCCCATGCAGAGCAAA-3′ |
| **CrPMK** | 5′- GCAGATGTCCAGGGAGACGATGT-3′ | 5′- ACATGATCGCGCAGACAGCC-3′ |
| **CrMVD** | 5′- GAAGGGCATCAAGATCACGAAGA-3′ | 5′- ATCCTCATCGCCGTGGTGTCCTC-3′ |
| **OsHMGS1** | 5′- GCCTACGCCTTCCTCCCAAT-3′ | 5′- GCCCACCTGCGTGCTCCAGGA -3′ |
| **OsHMGS2** | 5′- GGGATGGACGCTACGGTCTT-3′ | 5′- TAGCAGCAGCACCACCTGTT-3′ |
| **OsHMGS3** | 5′- TTGTTGCCTCCTGGGACGTT-3′ | 5′- GATCTCCTCGTCGGCCTTCC-3′ |
| **OsHMGR1** | 5′-GCTCTTGCCTCTGGTCACCT-3′ | 5′- GATGCGAAGGGCTGCTCAAG-3′ |
| **OsHMGR2** | 5′- CTCAGCTTCTTCGGCATCGC-3′ | 5′- GAAGTCCTCCTCCTCCTCCTC-3′ |
| **OsHMGR3** | 5′- GCGTGCAGAATGTGCTGGAT-3′ | 5′- CGCGCCCTTCGATCCAATTC-3′ |
| **OsMK** | 5′- CACTAGTGGATCGCGACCGT-3′ | 5′- TGGCCTGCACAATGCAAACT-3′ |
| **OsPMKa** | 5′-GTTAAGTTTTCCTTGCCTCC-3′ | 5′- TGCCACTGTTTCACAGATCC-3′ |
| **OsPMKb** | 5′- GACATGGTTAAATAAGTGCT-33′ | 5′- ACATTTCAATAGTTACAAGC-3-3′ |
| **OsMVD1** | 5′- TGCTATGACAGAGGAACAGC′-33′ | 5′- TTCCATCATTATTCTGTAAG-33′ |
| **OsMVD2** | 5′- AGTGGGATGCGAGACAGTGT-33′ | 5′- GAGACTTGATAGCCTCTTCC-33′ |
| **OsDXS1** | 5’-CTCAAGGGAGGGAAGAACAA-3’ | 5’-ACACCTGCTTGTTGTCGTTG-3’ |
| **OsDXS2** | 5’-TGTTGTGGAGCTCGCTATTG-3’ | 5’-TCCTCCCACCTAGATCCCTT-3’ |
| **OsDXS3** | 5′- ACCTCCTCGGGAAGAAGAAC-3′ | 5′- GAGGGACACCTGCTTGTTGT-3′ |
| **OsDXR** | 5′- GGTGACCTCTGAGCAATA-3′ | 5′- TCCACCCACAATCTATCA-3′ |
| **OsIPPI1** | 5’-AATGCTGCTCAGAGGAAGC-3’ | 5’-GGACGATGAACAGCAGGTA-3’ |
| **OsIPPI2** | 5′-GCAGACCTTTGCTGAAGTAAC-3′ | 5′-TTTAGTGGACGAACAGGACA-3′ |
| **OsHDR1** | 5′-GCTGAATGAGAAGAAGGTGC-3′ | 5′-AAAGGAAGCAGTGGCAAC-3′ |
| **OsHDR2** | 5′-GAGTAGGCGTTGTTGTGAATCAAAC-3′ | 5′-CCTTTCCTGAGTAGCATTGC-3′ |
| **OsHDS** | 5′-GGGTTGCATTGTCAATGG-3′ | 5′-CTGAATCAAGGCGTCAGT-3′ |
| **OsMDS** | 5′-TCGTCGGTGTTCATGAGG-3′ | 5′-TTGTCTCCTTGAATGGGC-3′ |
| **OsMCT** | 5′-GACTTGAGGTCACTGATG-3′ | 5′-AGCAAGTCATCATCAGGAGTC-3′ |
| **OsCMK** | 5′-ATGATGATGACTACAAGG-3′ | 5′-TCAGTCAGAAACTGATGC-3′ |
| **OsActin** | 5′- CATCGCCCTGGACTATGACC-3′ | 5′-CGGTGTGAGCTGCACGAACGA-3′ |

Table S1. PCR and RT-PCR primer list.


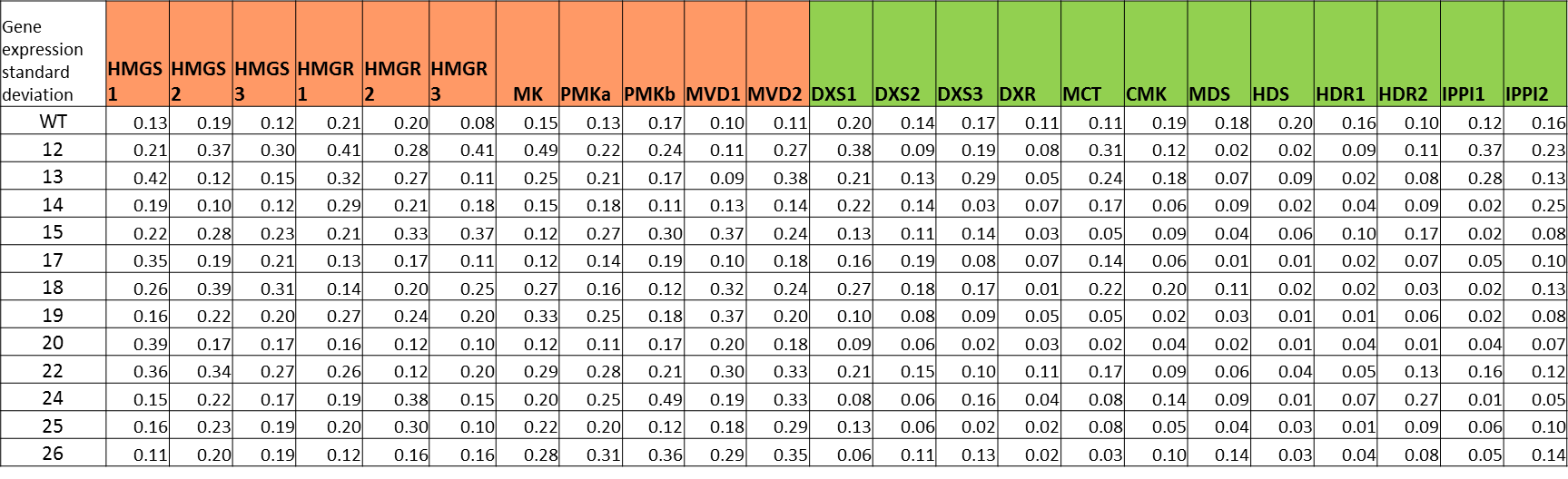


Table S2. Standard deviations of gene expression levels determined by real-time PCR in T2 seeds of transgenic plants expressing an ectopic MVA pathway compared to wild-type controls. Orange columns represent the endogenous MVA pathway and green columns represent the endogenous MEP pathway.

| **Gene isoform/Splicing** | **Accession Number** |
| --- | --- |
| OsHMGS1 | XM_015776782.2 |
| OsHMGS2 | XM_015794629.2 |
| OsHMGS3 | XM_015757147.1 |
| OsHMGR1 | XM_015768351.2 |
| OsHMGR2 | XM_015756838.2 |
| OsHMGR3 | XM_015792764.2 |
| OsMK | XM_015759462.2 |
| OsPMKa | XM_015777036.2 |
| OsPMKb | XM_015777037.2 |
| OsMVD1 | XM_026023121.1 |
| OsMVD2 | XM_015771206.2 |
| OsDXS1 | Os05g0408900 |
| OsDXS2 | Os06g0142900 |
| OsDXS3 | Os07g0190000 |
| OsDXR | Os01g0106900 |
| OsMCT | Os01g0887100 |
| OsCMK | Os01g0802100 |
| OsMDS | Os02g0680600 |
| OsHDS | Os02g0603800 |
| OsHDR1 | Os02g0793900 |
| OsHDR2 | Os03g0732000 |
| OsIPPI1 | Os07g0546000 |
| OsIPPI2 | Os05g0413400 |

Table S3. Accession numbers of the known isoforms of endogenous rice MVA and MEP pathway genes and the alternative splicing variants of *OsPMK*.

|  | **tZ** | **SA** | **JA** | **IAA** | **GA4** |
| --- | --- | --- | --- | --- | --- |
| **ACC** | 39% (–0.66) | 67% (0.63) |  |  |  |
| **ABA** |  | 52% (0.76) | 49% (0.82) |  |  |
| **GA4** |  |  |  | 36% (0.64) |  |
| **ZR** |  |  |  | 37% (0.65) | 51% (0.74) |

Table S4. Pairs of phytohormones showing significant correlations in abundance in the MVA+WR1 transgenic plants. For the phytohormones in each row, we generated linear models to calculate the percentage of variation of abundance that can be explained by variations in the abundance of the phytohormones heading each column. All effects below 20% or below statistical significance were omitted. Numbers between parentheses indicate the Spearman rank correlation between the levels of the two hormones.

**Supplementary Figures**


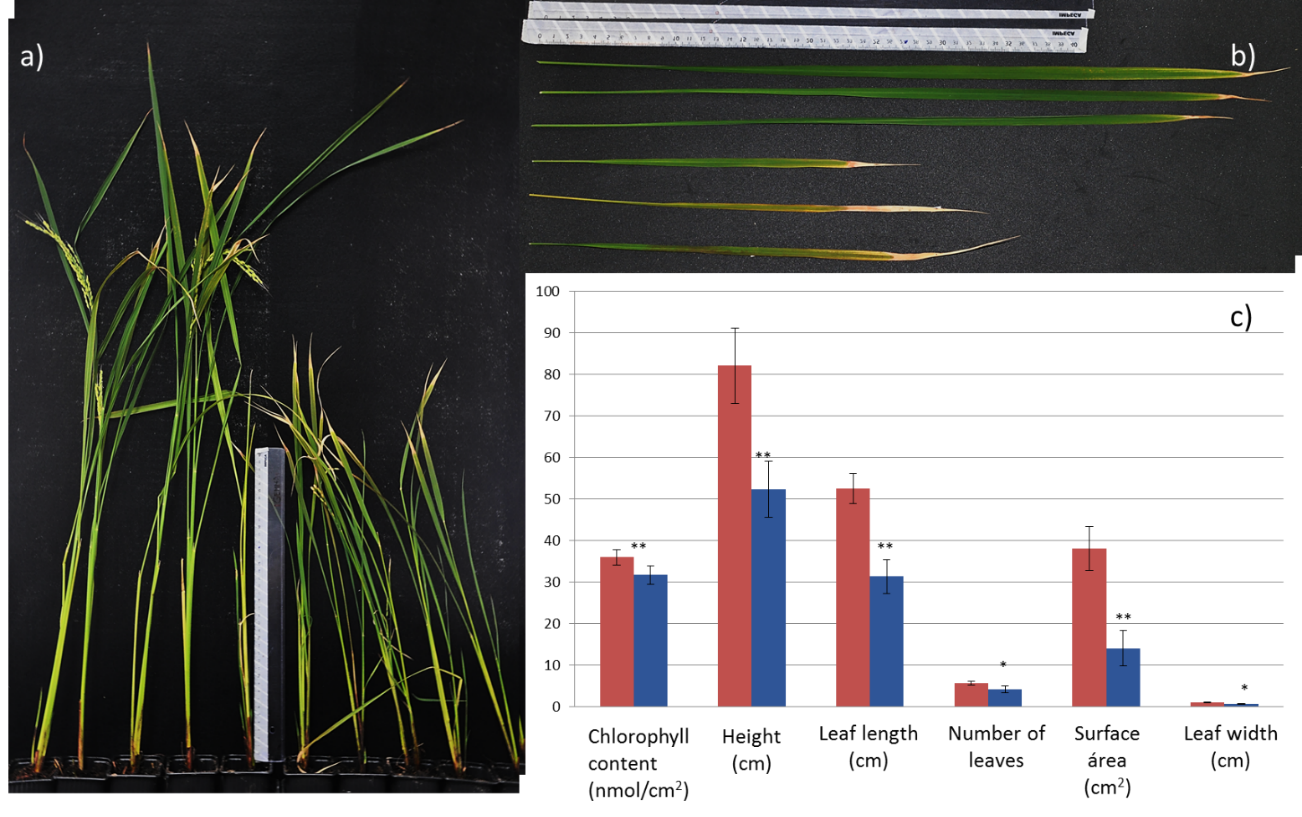


Supplementary Figure S1. Phenotypic analysis of transgenic rice plants. a) Five wild-type and five independent transgenic plants expressing MVA+WR1 (lines 14, 15, 18, 19 and 25, reflecting a range of transgene expression levels). b) Leaf phenotype of three wild-type and three independent transgenic plants expressing MVA+WR1 (lines 14, 18 and 19). c) Phenotypic analysis of wild-type and transgenic plants expressing MVA+WR1 . Values are means ± SD (n = 10 biological replicates with n = 3 technical replicates each, except chlorophyll content with n = 6 technical replicates). The asterisks indicate a statistically significant difference between wild-type and transgenic plants as determined by a Student’s t-test (*P < 0.05; **P < 0.01).


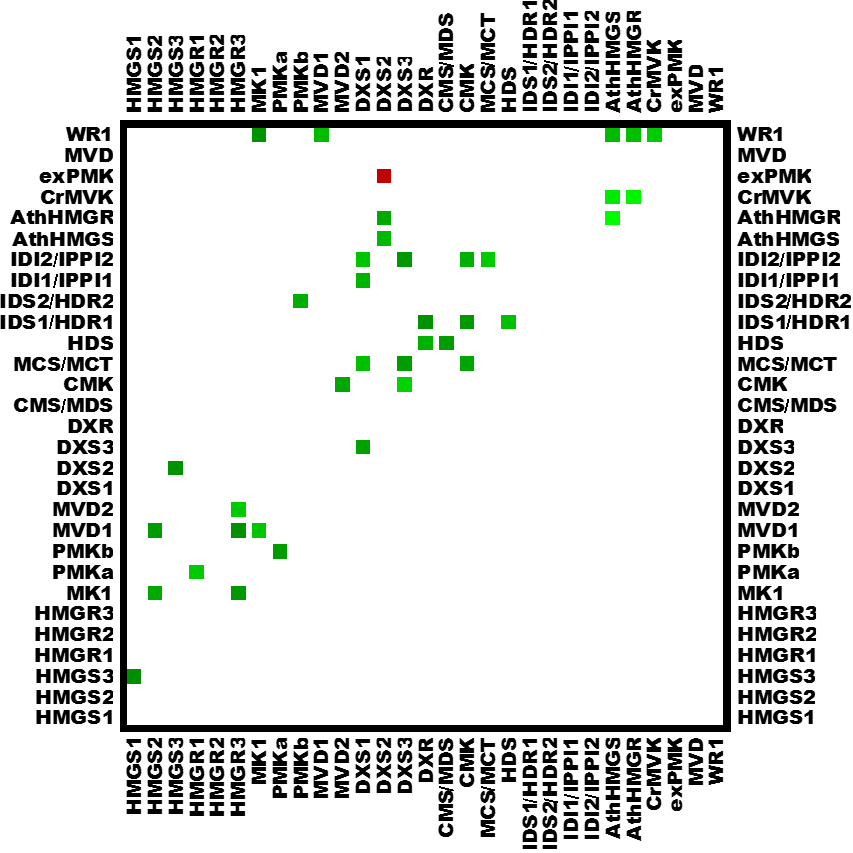


Supplementary Figure S2. Spearman correlation between endogenous gene expression levels in MVA+WR1 lines. Green squares indicate a positive correlation >0.5 between the expression levels of two relevant genes. Red squares indicate a negative correlation >0.5 between the expression levels of two relevant genes. Deeper color indicates an adjusted R^2^ close to 1. Overall, the expression of gene isoforms tends to be either uncorrelated or positively correlated.


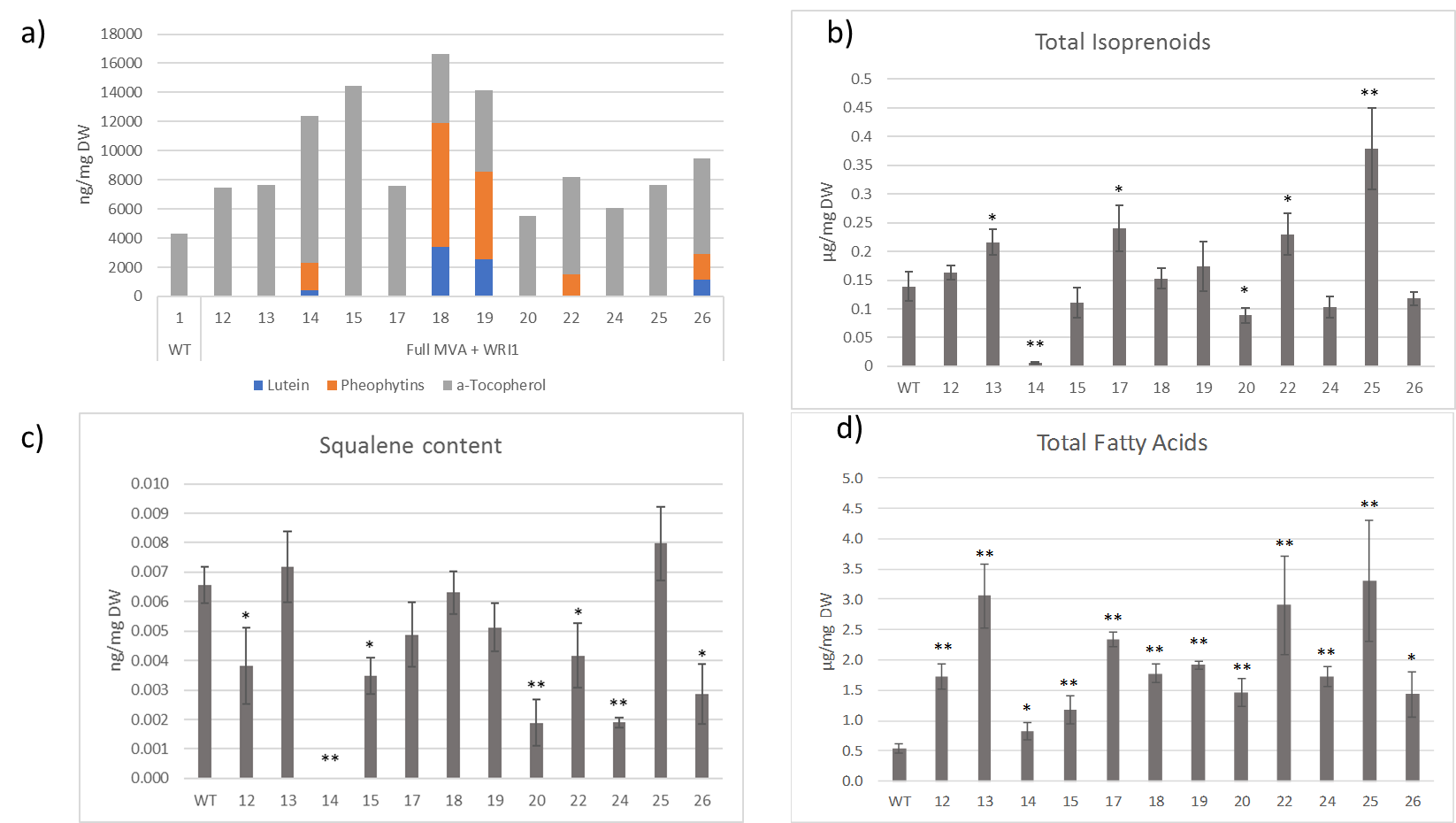


Supplementary Figure S3. UPLC and GC-MS analysis of metabolites in transgenic plants. a) UPLC analysis of lutein, α‑tocopherol and pheophytin in dry rice seeds. Wild-type (WT) seeds contained ~4000 ng mg^-1^ of tocopherol. b–d) GC-MS analysis of fatty acids, total isoprenoids and squalene in dry rice seeds. Values are means ± SD (n = 3 technical replicates). The asterisk indicates a statistically significant difference between WT and transgenic lines, as determined by Student’s t test (*P<0.05, **P<0.001).


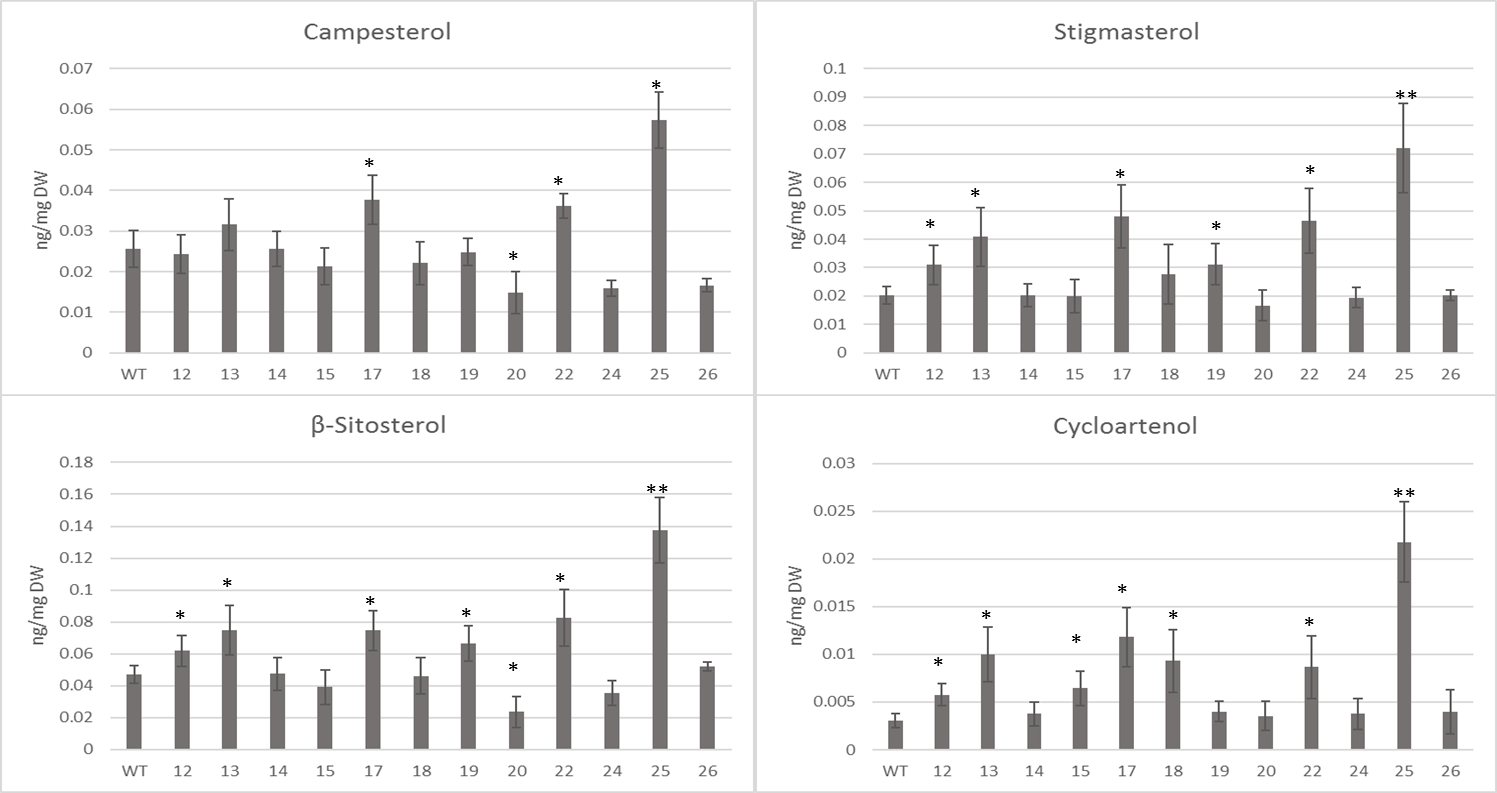


Figure S4. GC-MS analysis of campesterol, stigmasterol, β-sitosterol and cycloartenol levels in dry rice seeds. Values are means ± SD (n = 3 technical replicates). The asterisk indicates a statistically significant difference between wild-type (WT) and transgenic lines, as determined by Student’s t test (*P<0.05, **P<0.001).

Supplementary Figure S5. Seed and leaf carbohydrate content of wild-type and MVA-WR1 plants. Values are expressed as means ± SD (n = 10 biological replicates, each with n = 2 technical replicates). Asterisks indicate a statistically significant difference between WT and transgenic lines, as determined by Student’s t-test (*P < 0.05; **P < 0.01).


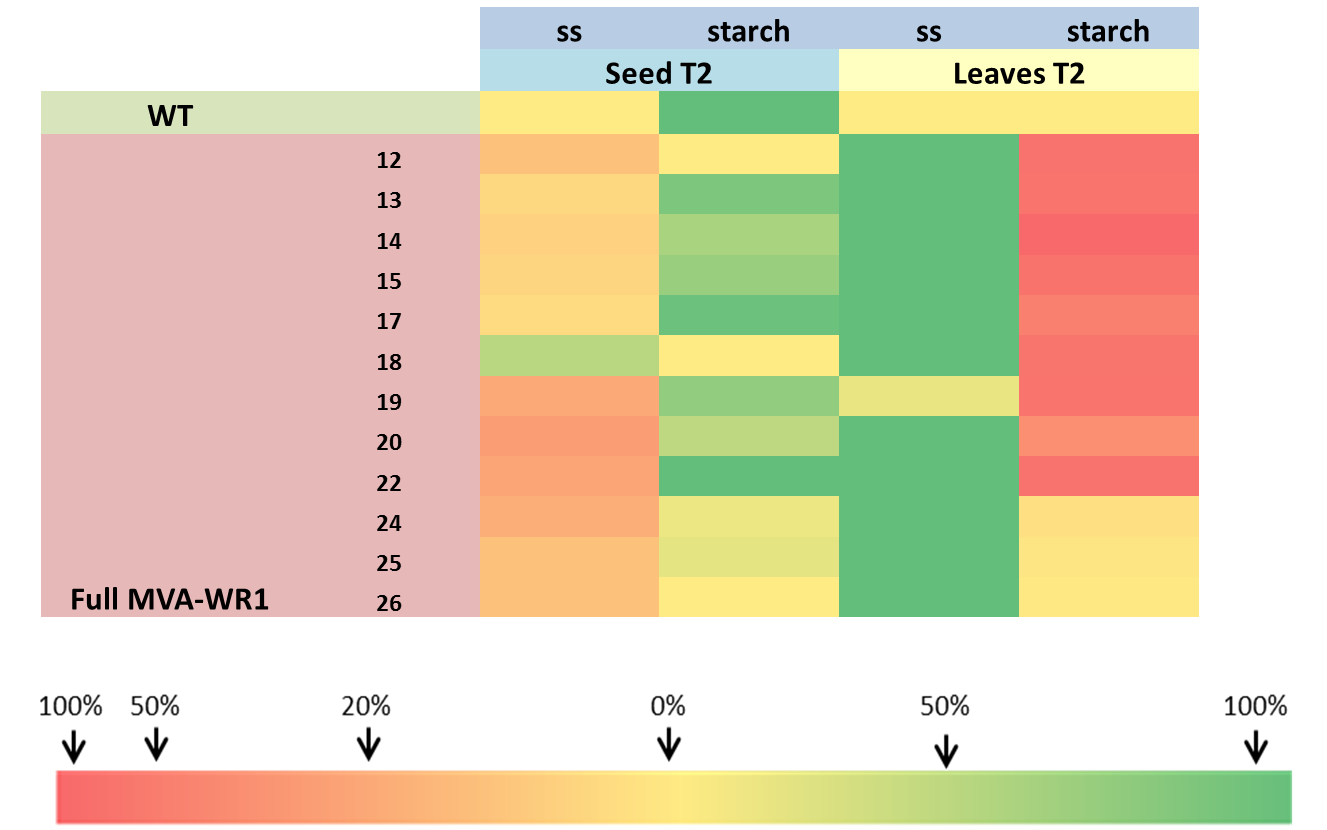


Figure S6. Heat map showing changes in starch and soluble sugar levels as a percentage relative to wild-type levels in the T2 seeds and leaves of transgenic lines. The red gradient shows decreasing levels and the green gradient shows increasing levels, with yellow indicating no change. The red gradient is expanded in the lower ranges because this is where most of the values lie, whereas the green gradient is linear.
